# Treatment Alternatives to Negotiate Peri-Implantitis

**DOI:** 10.1155/2014/487903

**Published:** 2014-06-15

**Authors:** Eli E. Machtei

**Affiliations:** Department of Periodontology, School of Graduate Dentistry, Rambam Health Care Campus and Faculty of Medicine, Technion (Israel Institute of Technology), Rambam HCC, 8 Ha'alia Hashnia Street, 31096 Haifa, Israel

## Abstract

Peri-implant diseases are becoming a major health issue in dentistry. Despite the magnitude of this problem and the potential grave consequences, commonly acceptable treatment protocols are missing. Hence, the present paper reviews the literature treatment of peri-implantitis in order to explore their benefits and limitations. Treatment of peri-implantitis may include surgical and nonsurgical approaches, either individually or combined. Nonsurgical therapy is aimed at removing local irritants from the implants' surface with or without surface decontamination and possibly some additional adjunctive therapies agents or devices. Systemic antibiotics may also be incorporated. Surgical therapy is aimed at removing any residual subgingival deposits and additionally reducing the peri-implant pockets depth. This can be done alone or in conjunction with either osseous respective approach or regenerative approach. Finally, if all fails, explantation might be the best alternative in order to arrest the destruction of the osseous structure around the implant, thus preserving whatever is left in this site for future reconstruction. The available literature is still lacking with large heterogeneity in the clinical response thus suggesting possible underlying predisposing conditions that are not all clear to us. Therefore, at present time treatment of peri-implantitis should be considered possible but not necessarily predictable.

## 1. Introduction

Peri-implantitis is becoming an ever growing oral health concern that is frequently encountered in the dental office. The number of dental implants that are currently placed annually is somewhat elusive; however, the best estimate available puts this figure at around fifteen million new implants (worldwide) every year [1]. Of these, how many will eventually develop peri-implant diseases is also debatable. Zitzmann and Berglundh on behalf of the VI workshop of the European Federation of Periodontology have suggested that 80 percent of the patients and 50 percent of the implants will develop peri-implant mucositis during the years. These corresponding figures for peri-implantitis are 28–56 percent of the patients and 12–43 percent of the implants [2]. To the contrary, Mombelli et al., on behalf of the 3rd European academy of osteointegration workshop in 2012, have suggested somewhat lower numbers for peri-implantitis: 20 percent of the subjects and 10% of the implants [3]. More recently, Atieh and coworkers [4] in a meta-analysis of 504 studies which included 1497 patients with 6293 implants reported the prevalence of peri-implant mucositis to be 63.4 percent (of the patients) and 30.7 percent (of the implants). A higher frequency of occurrence of peri-implant diseases was recorded for smokers with a summary estimate of 36.3 percent.

The reason for this large variation in the reported literature might be associated with patients variables such as smoking [5, 6], preexisting periodontal disease [7, 8], oral hygiene [9, 10], quality of prosthetic reconstruction [11, 12], and some systemic conditions and medications [13, 14]. Koldsland and coworkers [15] have suggested a different approach to explain this variability. Using different threshold levels to define peri-implantitis (i.e., bone loss of >2 mm or >3 mm and implants probing depth of >4 mm and >6 mm), the prevalence of peri-implantitis varied significantly from 11.3 to 47.1 percent. Thus, with the current lack of universally accepted criteria for the definition of peri-implantitis, the use of different thresholds is likely to produce different prevalence rates. However, even with the more conservative estimates, the number of new implants that are likely to be affected by peri-implant diseases every year is the high million range, of which 7-8 million are likely to develop peri-implant mucositis while about 3-4 million will develop peri-implantitis.

**Figure 1:**
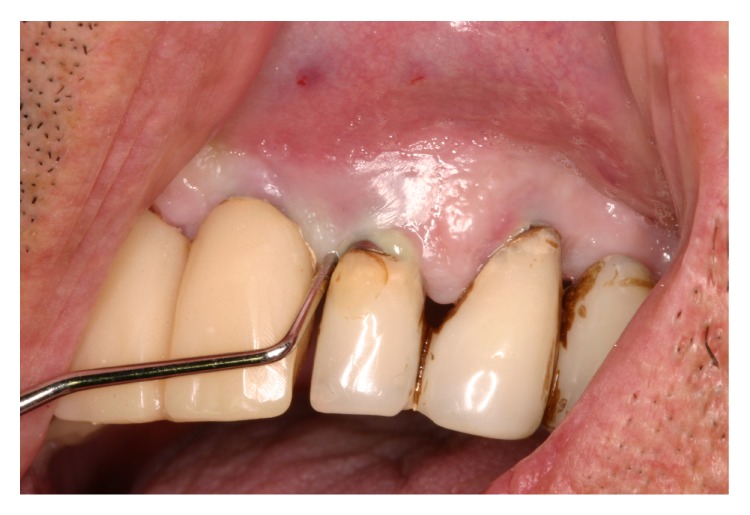
Peri-implantitis with suppuration.

Nevertheless, despite the magnitude of this problem and the potential grave consequences that may result from a nonresponsive peri-implantitis condition, a commonly acceptable treatment protocols are yet to be agreed upon. Hence, the purpose of this paper is to review the available literature pertaining to both surgical and nonsurgical therapies of peri-implantitis in order to explore their benefits and limitations (Figure 1).

## 2. Body 

The treatment of peri-implantitis may include each of the following modalities, either individually or combined: nonsurgical therapy is aimed at removing local irritants from the implants' surface with or without surface decontamination and possibly some additional adjunctive therapies. Surgical therapy is aimed at removing any residual subgingival deposits and additionally reducing the peri-implant pockets depth. This can be done alone or in conjunction with either osseous respective therapy or the contrary regenerative approach. Finally, if all fails, explantation of the affected nonresponsive implant might be the best alternative in order to arrest the destruction of the osseous structure around the implant, thus preserving whatever is left in this site for future reconstruction.

### 2.1. Nonsurgical Treatment of Peri-Implantitis 

#### 2.1.1. Implant's Debridement

The microbial origin of peri-implantitis has been previously established [16]. Shibli et al. [17] in an experimental peri-implantitis study in the canine model have found* Prevotella intermedia/nigrescens* in 13.89% of implants at baseline and 100% of implants at other periods.* Porphyromonas gingivalis* was not detected at baseline, but after 20 and 40 days it was detected in 33.34% of implants and at 60 days it was detected in 29.03% of dental implants.* Fusobacterium *spp. were detected in all time points. Streptococci were detected in 16.67% of implants at baseline and in 83.34%, 72.22%, and 77.42% of implants at 20, 40, and 60 days, respectively.* Campylobacter *spp. and* Candida *spp. were detected in low proportions. Total viable count analysis showed significant difference after ligature placement (*P* < 0.0014). However, there was no significant qualitative difference, in spite of the difference among the periods. The same authors (2008) have compared the microbial biofilm of healthy and peri-implantitis implants; they found significantly higher mean counts of* Porphyromonas gingivalis*,* Treponema denticola*, and* Tannerella forsythia* in the peri-implantitis sites, both supra- and subgingivally. Also, the proportions of the pathogens from the red complex were elevated, while host-compatible beneficial microbial complexes were reduced in diseased compared with healthy implants. The microbiological profiles of supra- and subgingival environments did not differ substantially within each group [18]. Thus, the need for implant debridement in order to eliminate bacterial flora that is likely associated with the peri-implant disease is obvious.

However, the clinical efficacy of this treatment modality has been shown to be relatively limited. Persson et al. [19] in a single-blinded randomized longitudinal clinical study of mechanical nonsurgical treatment of peri-implantitis reported that the most prevalent bacteria were* Fusobacterium nucleatum *sp.,* Staphylococcus* sp.,* Aggregatibacter actinomycetemcomitans*,* Helicobacter pylori*, and* Tannerella forsythia*. 30 min after treatment (with curettes only),* Aggregatibacter actinomycetemcomitans* (serotype a),* Lactobacillus acidophilus*,* Streptococcus anginosus*, and* Veillonella parvula* were found at lower counts (*P* < 0.001). However, at 6 months, microbiological differences between baseline and 6-month samples were not significant for any species or between treatment methods in these peri-implantitis sites. Renvert et al. in a corresponding clinical report of this study [20] have shown minimal pocket reduction between baseline (5.1 mm) and 6-month (4.9 mm) measurements; *P* = 0.30. These minimal changes were attained in both groups (ultrasonic instruments and the hand held curettes). Plaque scores at treated implants decreased from 73% to 53% (*P* < 0.01). Bleeding scores also decreased (*P* < 0.01), again with no group differences.

#### 2.1.2. Surface Decontamination

To try improving the outcome of nonsurgical therapy of peri-implantitis site, the use of surface decontamination has been studied extensively. This is usually being performed mechanically via the use of air abrasive devices or chemical agents.

*In vitro* studies have confirmed the potency of this device to remove plaque and biofilm. Tastepe et al. [21] have studied the cleaning and modification of intraorally contaminated medium roughness titanium discs using calcium phosphate powder abrasive treatment. They have concluded that air powder abrasive methods using various agents were all efficient in removing the biofilm from contaminated titanium discs. Nonetheless, clinical studies of the efficacy of this treatment approach had produced mixed results. In a recent prospective, randomized, controlled clinical study, Sahm and coworkers [22] compared the efficacy of nonsurgical treatment of peri-implantitis using an air abrasive device versus mechanical debridement and local application of chlorhexidine solution. After six months, the air abrasive group revealed significantly higher changes in mean BOP scores when compared with mechanically treated sites (43.5 ± 27.7% versus 11.0 ± 15.7%). However, pocket reduction was minimal (0.6 mm) in both treatment groups. Likewise, clinical attachment level gains were minimal and very similar in both groups (0.4 ± 0.7 mm and 0.5 ± 0.8 mm, resp.). Likewise, Renvert et al. [23] in a nonsurgical treatment study of peri-implantitis patients have reported a mean pocket reduction after 6 months to be 0.9 mm following this intervention. While this seems to be slightly greater than that reported for debridement only, still the magnitude of these changes is not sufficient. Thus, none surprisingly, Tastepe et al. [24] in a recent review of the literature have concluded that the* in vivo* data on air powder abrasive treatment as an implant surface cleaning method is not sufficient to draw definitive conclusions.

#### 2.1.3. The Use of Lasers

The efficacy of different laser wavelength to eliminate bacteria from implants' surface had been demonstrated* in vitro*. Deppe and coworkers [25] used a XeCl 308 nm excimer laser irradiation with a constant energy of 0.8 J/cm and a constant frequency of 20 Hz on peri-implantitis-associated bacteria* in vitro*. They have been able to show that 200 pulses were sufficient to reduce the replication of these anaerobic microorganisms for more than 99.9%. Likewise, Kreisler and coworkers [26], using an Er:YAG laser on different implant surfaces contaminated with* Streptococcus sanguinis*, were able to report that, compared to nonirradiated specimens, mean bacterial reductions ranged from 99.51% to 99.6% at a pulse energy of 60 mJ and from 99.92% to 99.94% (TPS) at 120 mJ. The adjunctive effect of photodynamic therapy in conjunction with soft laser therapy was also studied* in vitro* by Haas et al. [27]. After contaminating these rough surface implants with* Actinobacillus actinomycetemcomitans* or* Porphyromonas gingivalis* or* Prevotella intermedia*, these surfaces were then treated with a toluidine blue solution and irradiated with a diode soft laser with a wave length of 905 nm for 1 min. None of the smears obtained from the thus treated surfaces showed any bacterial growth, whereas the smears obtained from the controls showed unchanged growth of every target organism tested. Likewise, Salmeron et al. [28] in a preclinical rat model have studied laser therapy alone or with photodynamic therapy and compared them to both negative and positive controls for implant surface decontamination. The results of this histomorphometric study were then followed longitudinally: while photodynamic therapy showed some improved early (7 days) results; over longer time periods (>14 days), all methods produced similar results.

Here again, clinical studies have failed to support the* in vitro* microbiological results. Renvert [23] in his clinical study which explored different treatment modalities for peri-implantitis reported minimal (0.8 mm) pocket reduction around implants treated with Er:YAG laser. Likewise, Schwarz et al. [29] in a clinical and histological study using Er:YAG laser in peri-implantitis patients reported that while patients exhibited some improvements in the clinical parameter this amounted to approximately 0.5 mm for all time intervals (up to 24 months); furthermore, histopathological examination of tissue biopsies revealed a mixed chronic inflammatory cell infiltrate (macrophages, lymphocytes, and plasma cells) which seemed to be encapsulated by deposition of irregular bundles of fibrous connective tissue showing increased proliferation of vascular structures. Thus, they have concluded that a single course of nonsurgical treatment of peri-implantitis using ERL may not be sufficient for the maintenance of failing implants. These authors in yet another study [30] reported that mean value of BOP decreased in the Er:YAG treated group from 83% at baseline to 31% after 6 months while in the C group from 80% at baseline to 58% after 6 months. The sites treated with Er:YAG demonstrated a mean CAL change from 5.8 ± 1 mm at baseline to 5.1 ± 1.1 mm after 6 months; similarly, the C sites demonstrated a mean CAL gain from 6.2 ± 1.5 mm at baseline to 5.6 ± 1.6 mm at 6 months (the difference between the two groups being statistically insignificant). Most recently, Esposito and coworkers [31] in a one-year multicenter pragmatic randomized controlled clinical trial of the adjunctive use of light-activated disinfection (LAD) in the treatment of peri-implantitis have concluded that LAD therapy (FotoSan) with mechanical cleaning of implants affected by peri-implantitis did not improve any clinical outcomes when compared to mechanical cleaning alone up to 1 year after treatment.

#### 2.1.4. The Adjunctive Effect of Local Delivery of Antibacterial Agents

To further improve the response to nonsurgical treatment of peri-implantitis, the use of local delivery of antibacterial agents has been advocated. As early as 2001, Mombelli and coworkers [32] explored the adjunctive effect of tetracycline fibers in the nonsurgical treatment of peri-implantitis. After twelve months, a significant decrease in frequency of detection was noted for* Prevotella intermedia/nigrescens*,* Fusobacterium *sp.,* Bacteroides forsythus*, and* Campylobacter rectus*. Clinically, mean pocket reduction was 1.2–1.9 mm which was maintained up to 12 months postop. However, three subjects have shown continuous deterioration in the clinical parameters and were thus removed from the study and are not included in the results.

Renvert and coworkers [33] in a 12-month clinical study of 32 patients with peri-implantitis were treated with minocycline microspheres or chlorhexidine gel as adjunct to mechanical debridement: At one year, pocket reduction amounted to only 0.6 mm with no difference between the two treatment groups. Salvi and coworkers [34] in a similar study reported some greater pocket reduction (1.6 mm) in implants treated with minocycline microspheres. However, 6 implants in six subjects (of the original 31 implants) required rescue treatment or were exited from the study all together, due to continuing attachment level loss despite this treatment. More recently, Schär et al. [35] reported that, 3 months following treatment with either minocycline microspheres of photodynamic therapy, implants of both groups yielded a statistically significant reduction in the number of BOP-positive sites compared with baseline. Changes in implants probing depth, while statistically significantly different from baseline, amounted to only 0.4 mm. One should keep in mind that the initial pocket depth in these sites was moderate. Likewise, CAL gain was approximately 0.25 mm.

The use of chlorhexidine irrigation was studied in a preclinical canine study by Porras et al. [36]. Subjects received dental prophylaxis and were randomly assigned to the control group (mechanical debridement and oral hygiene instructions) or to the test group (antiseptic therapy which included mechanical cleansing and oral hygiene instructions supplemented by local irrigation with chlorhexidine 0.12%, using a plastic syringe, and topical application of a 0.12% chlorhexidine gel). Both treatment modalities were effective in reducing peri-implant infection and implants probing depths and in improving attachment levels with no intergroup differences. Similarly, Sahm and coworkers [22] in a clinical trial of nonsurgical treatment of peri-implantitis sites reported that implants' mean pocket reduction was 0.8 mm and attachment level gain was also 0.8 mm when using mechanical debridement and adjunctive subgingival irrigation with CHX solution and gel application into the pockets. Likewise, Renvert et al. [37] have used chlorhexidine gel in conjunction with mechanical debridement for the treatment of moderate pocket sites around dental implants. Only moderate pocket reduction (0.43 mm) could be attained; however, bleeding on probing was significantly reduced from 86% of the sites at screening to 30% at the end of the study, one year later.

Büchter and coworkers [38] in a randomized clinical trial were using doxycycline gel in the peri-implant pockets as an adjunct implants mechanical therapy. Pocket reduction (1.15 mm) and CAL gain (1.17 mm) were significantly greater than those of the mechanical treatment only (0.56 mm for both outcomes).

More recently, our group has reported in a randomized double blind placebo controlled multicenter clinical trial on the use of chlorhexidine containing chips (Periochip) in the treatment of peri-implantitis [39]. In this study of moderate to severe peri-implantitis sites, chlorhexidine containing chips were repeatedly inserted into the peri-implant pockets every other week (unless pockets were already reduced to 5 mm or less) for a period of up to 3 months. This novel approach of repeated placement of chlorhexidine chips has resulted in a significant improvement of the peri-implant soft tissue parameters six months postop: pocket reduction (mean 2.29 mm) and attachment level gain (2.21 mm) were significantly better than those previously reported for nonsurgical treatment of peri-implantitis. Furthermore, 73% of these sites had had pocket reduction of 2 mm or greater, while 40 percent had pocket reduction of at least 3 mm.

Conversely, Renvert et al. [40] in a similar nonsurgical treatment study of moderate peri-implantitis sites used repeated subgingival application of minocycline microspheres (Arestin) once a month for up to three months. Mean pocket reduction in the deepest sites amounted to 0.9 mm in the experimental group.

#### 2.1.5. Systemic Antibiotics

The use of systemic antibiotics as an adjunctive tool in nonsurgical periodontal therapy had been shown to have small but statistically significant added benefit over scaling and root planning alone [41]. Thus, the use of such protocols in the nonsurgical treatment of peri-implantitis would seem like a logical course to take. Hallström et al. [42] have compared, in a randomized clinical trial design, nonsurgical treatment of peri-implant mucositis with or without systemic antibiotics. Forty-eight subjects received nonsurgical debridement with or without systemic Azithromycin for four days and were followed during 6 months. Pocket reduction was 0.9 mm in the antibiotics group (1.4 mm in the deepest sites) and 0.5 mm in the debridement only group (0.8 mm in its deepest sites). However, these differences between the antibiotics and control group were not statistically significant.

Lindhe and Meyle [43] on behalf of the VI European workshop in periodontology have concluded that there was limited evidence that nonsurgical treatment of peri-implantitis with the adjunctive use of systemic antibiotics could resolve a number of peri-implantitis lesions. Most recently, Javed et al. [44] in a systematic review of the use of antibiotics in the treatment protocol of peri-implantitis concluded that the significance of adjunctive antibiotic therapy in the treatment of peri-implantitis remains controversial. A potential explanation for this minimal adjunctive effect for these systemic antibiotics comes from a recent study by Rams and coworkers [45]. In this study, a hundred and six peri-implantitis sites in 120 patients were sampled microbiologically and tested for potential antibiotic resistance. They found that one or more cultivable submucosal bacterial pathogens (most often* Prevotella intermedia/nigrescens *or* Streptococcus constellatus*) were resistant* in vitro* to clindamycin, amoxicillin, doxycycline, or metronidazole in 46.7%, 39.2%, 25%, and 21.7% of the peri-implantitis subjects, respectively. Overall, 71.7% of the 120 peri-implantitis subjects exhibited submucosal bacterial pathogens resistant* in vitro* to one or more of the tested antibiotics.

Another important issue that needs to be discussed* vis-a-vis* the use of systemic antibiotics for the treatment of peri-implantitis is the risk for antibiotic resistance as a worldwide health hazard. The wide spread use of antibiotics in medicine at large in the past fifty years is now back firing at our profession with the ever increasing prevalence of resistant bacterial strains [46, 47]. This phenomenon is causing a medical crisis that might have severe and far reaching repercussions on the population. Thus, the use of antibiotics should be restricted to patients and conditions where it has been clearly shown to have significant benefits which outweigh the risks that are involved [48, 49]. Thus, current research has not yet substantiated such benefits and consequently systemic antibiotics should be limited to acute phase of peri-implant infection rather than to be the treatment of choice for peri-implantitis [50].

### 2.2. Surgical Treatment of Peri-Implantitis

#### 2.2.1. Open Flap Debridement

The clinical scenario in humans differs significantly from that in animals. The greatest difference is the inability, in most cases, to remove the prosthetic super structure in order to allow for a submerged healing of the regenerated sites. Thus, the results of many of the human clinical trials are less favorable and more diverse than these reported in animals models. Still, open flap debridement is the treatment of choice by many clinicians. Lagervall and Jansson [51] in a retrospective study of treatment outcome in patients with peri-implantitis performed in a private practice based clinical setting have reported that open flap debridement was selected for 47 percent of the sites affected by peri-implantitis. Albouy et al. [52] in a preclinical experimental peri-implantitis study in canines have compared responses to open flap debridement surgery (as a stand-alone procedure) in four different implants design and surface topographies. Two of the four TiUnite implants were lost after surgical therapy. Radiographic bone gain occurred at implants with turned, TiOblast, and SLA surfaces, while at TiUnite implants additional bone loss was found after treatment. Resolution of peri-implantitis was achieved in tissues surrounding implants with turned and TiOblast surfaces. Thus, they concluded that resolution of peri-implantitis following treatment without systemic or local antimicrobial therapy is possible, but the outcome of treatment is influenced by implant surface characteristics. Similarly, Persson et al. [53] have studied the effect of implants surface topography on reosseointegration in an experimental peri-implantitis model in the canines. Implants with turned surface were compared with SLA implants when treated with systemic antibiotics followed by open flap debridement. Treatment resulted in a 72% bone fill of the bone defects at turned sites and 76% at SLA sites. The amount of reosseointegration was 22% at turned sites and 84% at SLA sites. Nonetheless, while these variations do exist, open flap debridement offers a useful tool to negotiate peri-implant disease. Máximo et al. [54] in a short term clinical study were able to show that three months following access flap surgery all clinical parameters have improved. For the peri-implantitis groups, mean reduction in CAL was 2.3 ± 1.6 mm and mean implants pocket reduction was 3.1 ± 1.7 mm. Levels of* Treponema denticola*,* Tannerella forsythia*, and* Parvimonas micra *and of* Fusobacterium nucleatum* were significantly reduced after peri-implantitis therapy. In addition, counts of* Porphyromonas gingivalis* and* Treponema socranskii *and the proportions of red complex were also reduced. These same authors in a subsequent report have shown that TNF-alpha levels, initially much greater than healthy controls, were significantly reduced achieving the same level as the healthy group at 3 months after therapy [55]. Mechanical therapies alone were effective in treating mucositis and peri-implantitis over a period of 3 months. The open debridement procedure showed clinical and microbiological benefits on the treatment of peri-implantitis and could be safely used as a standard control group for future studies.

#### 2.2.2. The Supplementary Use of Osseous Resection

The use of osteoplasty and/or ostectomy to allow for better adaptation of the surgical flap and thus further improve the surgical outcome has been studied extensively. de Waal and coworkers [56] reported on thirty patients (79 implants) with peri-implantitis that were treated with apically repositioned flap, bone recontouring, and surface debridement and decontamination with 0.12% chlorhexidine gluconate + 0.05% cetylpyridinium chloride or placebo. Nine implants in two patients in the placebo-group were lost due to severe persisting peri-implantitis. The test group showed a significantly greater reduction in bacterial load, but clinical improvement (i.e., bleeding, suppuration, implants pocket depth, and radiographic bone loss) was sizeable however similar in both groups.

Serino and Turri [57] reported on their two-year prospective clinical trial of thirty-one subjects (86 implants) treated for peri-implantitis using a surgical procedure based on pocket elimination and bone recontouring. Two years following treatment, 15 (48%) subjects had no signs of recurrent peri-implant disease; 24 patients (77%) had no implants with a probing pocket depth of 0.6 mm associated with bleeding and/or suppuration following probing. Nevertheless, 36 implants (42%) out of the original 86 had had persistent peri-implant disease despite this treatment. The proportion of implants that remained healthy following treatment was higher for those with minor initial bone loss (2–4 mm bone loss as assessed during surgery) compared with the implants with an initial bone loss of 0.5 mm (74% versus 40%). Among the eighteen implants with bone loss of 0.7 mm at baseline, seven were explanted.

#### 2.2.3. The Complementary Use of Regenerative Techniques (Figure 2)

As early as 1993, Grunder et al. [58], in ligature-induced peri-implantitis study in canines using guided tissue regeneration with a nonresorbable ePTFE membrane and comparing it to flap surgery alone, reported that there were no differences between any of the clinical parameters in both the control and experimental sites from the submerged and nonsubmerged groups. Histologic and histomorphometric analyses also revealed no significant differences between groups with regard to new bone formation. Likewise, Nociti et al. [59] in a similar animal model compared different membranes, with and without additional bone graft, to flap surgery only for the treatment of peri-implantitis. Their results showed that debridement alone as well as grafting alone had the same effect as did either membrane. To the contrary, Hurzeler and coworkers [60] reported in a similar canine study that guided bone regeneration procedures resulted in the greatest amount of new bone formation, followed by bone grafts alone, and flap debridement. In humans, Roos-Jansåker et al. [61] were able to show similar response to therapy (implants pocket reduction of 2.9–3.4 mm and new bone fill of 1.4-1.5 mm) for peri-implantitis sites treated with either bone grafts alone or bone grafts in conjunction with resorbable collagen membrane. This same group [62], in a subsequent 3-year follow-up report, has found that this improvement was maintained almost unchanged three years later. Aghazadeh et al. have attempted to compare autogenous bone to bovine derived xenograft for the treatment of peri-implantitis [63]. At 12 months, bovine derived xenograft provided more radiographic bone fill than autogenous bone; however, the success for both surgical regenerative procedures was limited. More recently, Wiltfang et al. [64] have reported significant bone fill in a twelve-month clinical trial in which peri-implantitis sites were treated with surface decontamination and regenerative flap surgery that included a 1 : 1 ratio of autogenous and xenogeneic bone graft. Mean radiographic bone fill amounted to 3.5 mm. Schwarz et al. [65] presented a case series where twenty-two patients with moderate peri-implantitis were randomly treated with access flap surgery and the application of nanocrystalline hydroxyapatite (NHA) or a natural bone mineral in combination with a collagen membrane (NBM + CM). Clinical parameters were recorded at baseline and after 12, 18, and 24 months of nonsubmerged healing. After two years, both groups revealed clinically important probing depth reductions (NHA: 1.5 ± 0.6 mm; NBM + CM: 2.4 ± 0.8 mm) and clinical attachment level gains (NHA: 1.0 ± 0.4 mm; NBM + CM: 2.0 ± 0.8 mm). However, these clinical improvements seemed to be better in the NBM + CM.

Sahrmann et al. [66] in a recent systematic review have concluded that complete fill of the bony defect using GBR seems not to be a predictable outcome. The mucosal health status is left unconsidered in most studies. Well-controlled trials are needed to determine predictable treatment protocols for the successful regenerative treatment of peri-implantitis using GBR technique.

A possible explanation to this diversity in clinical response to regenerative surgical treatment around dental implants was suggested by Schwarz et al. [67]. In this study, three types of osseous defects around dental implants with peri-implantitis were treated with bone graft and resorbable collagen membranes. The circumferential defects sites yielded significantly better response than these sites with dehiscence type defect. Thus, defects' anatomy might affect the outcome of these regenerative techniques. Nonetheless, regenerative approach to peri-implantitis may at time produce significant improvement in these sites. Froum et al. [68] reported on long-term follow-up of 51 consecutively treated peri-implantitis sites (using combination of platelet-derived growth factor with an organic bovine bone or mineralized freeze-dried bone coverage with a collagen membrane or a subepithelial connective tissue graft). Probing depth reductions at 3 to 7.5 years of follow-up were 5.1–5.4 mm. Concomitant bone level gain was from 3.0 to 3.75 mm. None of these implants lost bone throughout the duration of the study. Another source for this diversity in implants response to regenerative treatment could be associated with implants surface topography. Roccuzzo and coworkers [69] reported on twenty-six patients with one crater-like defect, around either TPS or SLA dental implants, with a probing depth (PD) of 0.6. Following flap approach, the implant surface was mechanically debrided and treated using a 24% EDTA gel and a 1% chlorhexidine gel and the osseous defect filled with a bovine-derived xenograft (BDX); all sites were left to heal in a nonsubmerged environment. At one-year follow-up mean implants pocket depth reduction was 2.1 ± 1.2 mm in the TPS implants compared to 3.4 ± 1.7 mm in the SLA group (these differences being statistically significant). Complete defect fill was never found around TPS group, while it occurred in three out of 12 SLA implants. Finally, submergence of the dental implants during the healing of the regenerative procedure might have a beneficial effect on the outcome. Roos-Jansåker and coworkers [70] reported on a one-year case series of twelve patients with a progressive loss of > or = 3 threads (1.8 mm) following the first year of healing. Following flap reflection, implants were decontaminated with 3% hydrogen peroxide and a bone substitute (Algipore) was grafted with a resorbable collagen membrane that was placed over the grafted defect and secured with the cover screw and covered by flaps for 6-month submerged healing; after that time, the abutment was reconnected to the suprastructure. At twelve months, implant probing depth was reduced by 4.2 mm and a mean defect fill of 2.3 mm was achieved. However, comparisons of human trials between submerged and nonsubmerged healing protocols are yet to be done. Nevertheless, Schwarz et al. [71] compared nonsubmerged and submerged healing of ligature induced peri-implantitis in 5 beagle dogs (30 implants). In this study, both treatment procedures resulted in statistically significant improvements of all clinical parameters at both nonsubmerged and submerged implants. However, radiological improvements were only observed at submerged implant sites. Histomorphometrical analysis revealed that all nonsubmerged implants exhibited low amounts of new BIC (1.0–1.2%), while mean BIC was statistically significantly higher in the respective submerged implant groups (8.7%–44.8%).

The best grafting material to be used in the surgical management of peri-implantitis is yet to be determined. Different studies have employed different materials; however, the diversity in clinical design, defect morphology, outcome variables, and follow-up period makes their comparison nearly impossible [66, 72–76]. One of the most likely regenerative materials that was least tested is autogenous bone graft. Romanos and Nentwig [77] reported on a comparative study of peri-implantitis sites treated with autogenous bone or a xenogeneic bone grafting material (BioOss) both covered with a collagen membrane. In this study, radiological resolution of the lesions was observed for most sites with no intergroup differences. Schou and coworkers used different decontamination agents and different regenerative materials for the treatment of experimental peri-implantitis in monkeys [78–80]. Autogenous bone alone was compared to autogenous bone plus membrane, membrane alone, or a conventional flap procedure alone as a negative control. The animals were sacrificed 6 months after treatment. Healthy peri-implant tissue was established irrespective of the applied surgical procedure. A mean bone gain of 4.7 mm was identified around implants treated with autogenous bone plus membrane, while 4.0 mm, 3.0 mm, and 1.9 mm of bone gain were recorded for the bone only, membrane only, and conventional flap only groups, respectively. Quantitative digital subtraction radiography confirmed considerable bone gain within defects treated with autogenous bone with or without membrane coverage. The bone gain, especially for defects treated with combined bone-membrane approach, seemed to be almost at the level before development of peri-implantitis. By contrast, 38 and 25% of the defect were on average characterized by bone gain when treated with membrane only or flaps only, respectively. Thus, the present studies demonstrate considerable bone regeneration after treatment of experimental peri-implantitis with autogenous bone graft particles in this monkey model.

Despite this ample piece of evidence showing good regenerative response with the use of regenerative materials, the superiority of this approach over a conventional open flap debridement is yet to be established. Khoshkam et al. [81] in a systematic review and meta-analysis aimed at evaluating the effectiveness of reconstructive procedures for treating peri-implantitis revealed that the weighted mean radiographic defect fill was 2.17 mm, probing depth reduction was 2.97 mm, clinical attachment level gain was 1.65 mm, and bleeding on probing reduction was 45.8%. Great variability in reparative outcomes was found attributed to patient factors, defect morphology, and reconstructive agents used. They have concluded however that, currently, there is a lack of evidence for supporting additional benefit of reconstructive procedures to the other treatment modalities for managing peri-implantitis.

#### 2.2.4. Systemic Antibiotics to Supplement Surgical Flap Approach

Systemic antibiotics as adjunct to peri-implant flap surgery treatment are commonly used. Heitz-Mayfield and coworkers [82] have recently reported on a prospective clinical trial of thirty-six implants in 24 partially dentate patients with moderate to advanced peri-implantitis that were treated using an anti-infective surgical protocol incorporating open flap debridement and implant surface decontamination, with adjunctive systemic amoxicillin and metronidazole. At twelve months, mean pocket reduction was 2.6 mm with all treated implants having a mean PD < 5 mm. 47% of the implants had complete resolution of inflammation with no bleeding on probing. 92% of implants had stable crestal bone levels or bone gain. There were no significant effects of smoking on any of the treatment outcomes. Leonhardt et al. [83] reported on a five-year clinical, microbiological, and radiological study into the treatment of peri-implantitis. Surgical exposure of the lesions and cleaning of the implants were performed using hydrogen peroxide. The patients were than given systemic antibiotics according to a susceptibility test of target bacteria that were previously cultured. The treatment was evaluated clinically, microbiologically, and radiographically at 6 months, 1 year, and 5 years. Seven out of 26 implants with peri-implantitis at baseline were lost during the 5-year follow-up period despite a significant reduction in the presence of plaque and gingival bleeding. Four implants continued to lose bone, 9 had an unchanged bone level, and 6 gained bone. Five of the patients were treated with antibiotics directed against putative periodontopathogens, that is,* A. actinomycetemcomitans*,* P. intermedia*, or* P. gingivalis*; three patients were treated for presence of enterics (*E. coli* and* E. cloacae*); and, in one patient, treatment was directed against* S. aureus*.

### 2.3. Explantation (Figure 3)

The management of peri-implantitis may at time be unpredictable especially for the more advanced lesion associated with severe bone loss [84, 85]. This may in turn lead to further bone loss, increase in pocket depth and suppuration, and consequently severe damage to the alveolar bone. Thus, explantation as a treatment option that will help arrest the progression of the destructive process is sometime advocated [86]. Moreover, severely compromised dental implants might be at greater risk for mechanical fracture [87] which may lead to further peri-implant bone loss. However, explantation of such compromised implant will require additional treatment to replace the now missing implants. Reimplantation of a new implant in the sites of the previously diseased implant is the most logical treatment option [88]; however, this treatment approach is not without limitation: bone loss that has occurred around the diseased implant might not allow for straightforward reimplantation. Instead, sometimes an elaborate augmentation procedure will be required before this site is ready for a redo implant placement [89]. Mardinger et al. [90] in a retrospective analysis of the factors affecting the decision to replace failed implants after they have been removed reported that the chances of a patient with minor bone loss undergoing reimplantation were 20 times greater (odds ratio, 20.4) than those of a patient with severe bone loss. The main patient-related reasons for avoiding reimplantation were the additional costs (27%), fear of additional pain (17.7%), and fear of a second failure (16.2%).

The survival and success rates of dental implants in previously failed implant sites were first reported by Alsaadi and coworkers [91]. A total of 41 patients (58 implants) experienced the nonintegration. Of those, seven implants (in seven subjects) have failed again (which represents a survival rate of 87.9%). Similarly, Grossmann and Levin (2007) reported on the success and survival of single dental implants placed in sites of previously failed implants [92]. Seventy five patients (with a total of 96 implants) experienced failure of one or more implants. Of those, 31 implants in 28 patients were replaced with a similar implant placed in the same location. Nine of the replacement implants failed, resulting in an overall survival rate of 71%. Follow-up ranged from 6 to 46 months. Replacement of maxillary and mandibular failed implants was similar. All failures occurred during the first year after implant replacement. In a similar retrospective study, we have shown that of fifty-six patients with a total of 79 redo implants that were followed for three years, thirteen implants failed and that resulted in an overall survival rate of 83.5%. Successful implants had greater diameter (4.05 ± 0.52 mm) than failed implants (3.72 ± 0.56 mm) did; however, these differences were only marginal (*P* = 0.06). Conversely, smoking habits, implants length and location, mode of placement, and spontaneous exposure did not have a significant effect on the outcome of this procedure [93]. Kim and coworkers [94] were able to report somewhat better results for redo of dental implants into previously contaminated sites where an implant was removed. The survival rate of the second implant after removal of failed implant was 88.3%. The marginal bone loss at the final (two years) follow-up was minimal (0.33 ± 0.49 mm). No significant difference in the failure rate of the second implant was observed between the immediate and delayed replacement groups (*P* > 0.05). This slightly greater survival rate the second time around (compare to other studies) might be attributed to the use of smoking (a strong confounding condition) as an exclusion criterion. Even slightly higher figure (92.3% CSR) was reported by Mardinger and coworkers [95] in a private practice based clinical study.

Alternatively, sites where previous implants were retrieved might be rehabilitated using fixed partial denture anchored to proximal implants, natural teeth, or combination of the above. The dogma of one implant per one missing tooth can no longer be supported automatically. Eliasson et al. [96] in an eighteen-year retrospective study of 123 implant patients have shown that survival rates for dental prostheses supported by 2 and 3 implants were 96.8% and 97.6%, respectively. Furthermore, the mean bone loss at 5 years was 0.3 mm for the two groups. No significant differences in bone loss (*P* > 0.05), implant failure rate (*P* > 0.05), or incidence of mechanical complications (*P* > 0.05) were found. More recently, Salvi and Brägger [97] in a systematic review concluded that the number of implants supporting an FDP was not associated with the prevalence of mechanical or technical complications nor with the implant survival or success rates. Likewise, the option of replacing a lost/removed implant with a 3-unit tooth-supported FPD is also a solid alternative. Pjetursson et al. [98] in a literature review and meta-analysis have reported that the five-year survival rate of tooth-supported FPD was 93.8% compared to 95.2% for an implant-supported FPD (with no statistical differences). The 10-year survival rates were 89.2% and 86.7% for teeth- and implant-supported prostheses, respectively (*P* > 0.05).

Another valid alternative will be to do a hybrid tooth-implant-supported fixed partial denture. In a systematic review, Weber and Sukotjo [99] have shown that, after an observation period of at least six years, implant survival and prosthetic success were similar for implant supported and tooth to implant supported prostheses. Likewise, Lang et al. [100] in their systematic review on the survival and complications of combined tooth-implant-supported FPD reported 90.1% implants' survival after 5 years and 82.1% after 10 years. The corresponding figures for the FPD survival were 94.1% and 77.8% after five and ten years, respectively. These results are very similar (both for survival and success) to what was reported for teeth-born and implants-born fixed prosthesis. Thus, such rehabilitation may be considered in cases where a potential abutment tooth is present across an edentulous site where one of the implants has failed.

Contrary to common beliefs, the use of nonrigid connection in such hybrid prostheses is not recommended. Nickening et al. [101] in a five-year follow-up of eighty-four hybrid fixtures have shown low rate of complications in these prostheses with rigid connection (5.3%) while these restorations with nonrigid connection exhibited significantly greater rate of complications (28.5%). Another risk associated with nonrigid connection increases the risk for intrusion of the abutment teeth [102].

## 3. Conclusions 

In the present review, we went through the literature pertaining to treatment alternatives to peri-implant diseases and the great diversity that is being reflected from this data. Both nonsurgical and surgical treatment strategies have shown to yield some beneficial effect on the peri-implant disease. However, while some implants/patients seemed to have benefited greatly from these treatment regiments, others have responded less favorably.

The most frustrating piece of information is the heterogeneity in the clinical response of peri-implantitis sites that were treated similarly as it was reported in the different studies.

Good independent randomized control trials are scarce, and the need for such well-designed studies was highlighted by Tonetti et al. [103] on behalf of the VIII European workshop in periodontology. Likewise, Esposito and coworkers [104] in a recent systematic review and meta-analysis that tried to identify the most effective interventions for treating peri-implantitis around osseointegrated dental implants have concluded that there is no reliable evidence suggesting which could be the most effective interventions for treating peri-implantitis.

We are still in the dark when it comes to the following questions.

- Which is the best decontaminating agent (or do we really need it all together)?
- Can nonsurgical therapies solve the mild to moderate peri-implantitis condition without a need to resort to access flaps?
- Are regenerative procedures superior to access flap only approach?
- Which of the regenerative techniques is most suitable in cases with peri-implantitis?

With the ever growing prevalence of peri-implant diseases, the need to address these questions is both real and urgent. Until that time when these data are available, treatment of peri-implantitis should be considered as possible but not necessarily predictable.

## Figures and Tables

**Figure 2:**
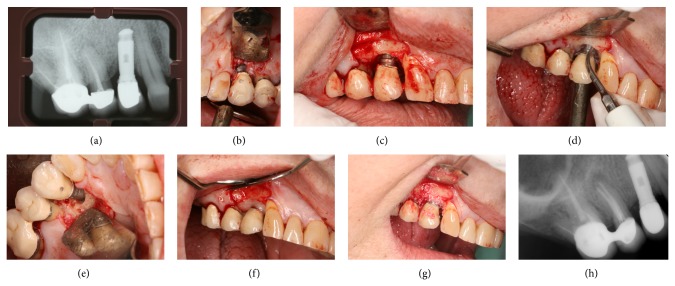
Treatment of peri-implantitis using a regenerative approach. (a) Preop, note the severe bone loss on implant at position #14. (b) Upon reflection of the flaps, note the granulation tissue but also excess cementum on the crown's margin. (c) Following degranulation, demonstrating the extent of bone loss. (d) Excess cement was removed and the implant surface was debrided using hand instruments and ultrasonic scaler. (e) Decortication was performed using diamond burs. (f) Surface decontamination was supplemented with the application of 24% EDTA for 3 minutes. (g) The defect was grafted with bovine derived Xenograft (BioOss). (h) 3 years later, complete resolution of the radiographic defect is evident.

**Figure 3:**
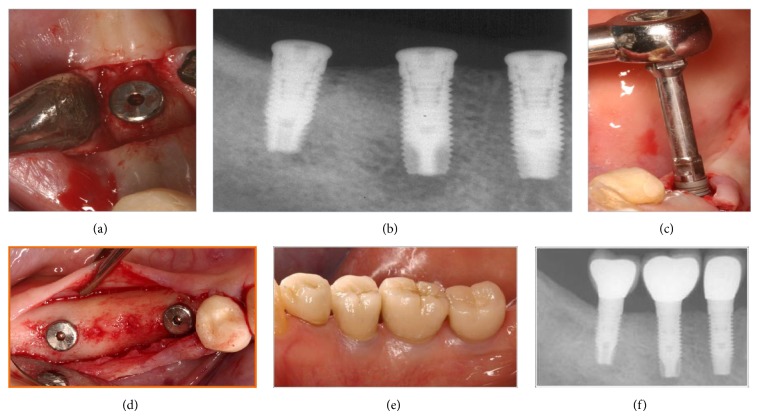
Explantation of dental implant.
